# Second-trimester uterine rupture in bicornuate uterus: A case report

**DOI:** 10.1016/j.crwh.2024.e00676

**Published:** 2024-12-14

**Authors:** Mesfin Ayalew Tsegaye, Zelalem Adugna Mekonnen, Dawit Takele Lemma, Alemayehu Nigusssie Adugna, Rebecca Haile Tesfay

**Affiliations:** Dilla University, College of Medicine and Health Sciences, Department of Obstetrics and Gynecology, Dilla, Ethiopia

**Keywords:** Uterine rupture, Second trimester, Mullerian anomalies, Bicornuate uterus, Hemoperitoneum

## Abstract

Uterine rupture is a rare but serious complication that predominantly occurs in the third trimester of pregnancy. It is exceptionally uncommon in the second trimester, particularly in the presence of uterine anomalies such as a bicornuate uterus or uterus didelphys. This case report presents a significant instance of second-trimester uterine rupture associated with a bicornuate uterus, resulting in a life-threatening intra-abdominal hemorrhage of approximately 4000 mL. The case report details the clinical presentation, diagnostic challenges, and management strategies used in this case, highlighting the critical importance of prompt recognition and intervention in similar scenarios to improve maternal outcomes.

## Highlights

• Uterine rupture is a rare occurrence that usually occurs in the third trimester and scarred uterus.
• Bicornuate uterus is a rare uterine anomaly.
• A case of second-trimester uterine rupture in bicornuate uterus was managed successfully.

## 1 Introduction

Uterine rupture is a rare but life-threatening complication that predominantly occurs in the third trimester of pregnancy. Its incidence in the second trimester is exceptionally low, particularly in the absence of pre-existing uterine scars. [1,2] Certain congenital uterine anomalies, such as a bicornuate uterus and didelphys uterus, have been identified as potential risk factors for uterine rupture [3]. Bicornuate uterus is a condition characterized by a double uterus with a single cervix and vagina and an external indentation of >1 cm, which can predispose patients to complications during pregnancy [4,5].

This report details a case of second-trimester uterine rupture in a 28-year-old woman (gravida 3, para 2) with a bicornuate uterus. The patient presented with alarming symptoms, including lower abdominal pain, vaginal bleeding, and marked abdominal distension, leading to a diagnosis of massive hemoperitoneum with an estimated blood loss of approximately 4000 mL. This report highlights the clinical presentation, diagnostic challenges, and management strategies employed, emphasizing the critical importance of timely intervention in similar situations to improve maternal outcomes. By sharing this case, we hope to raise awareness about the potential risks associated with uterine anomalies and the necessity for vigilance in monitoring pregnancies complicated by such conditions.

## 2 Case Presentation

A 28-year-old woman (gravida 3, para 2, both children alive and delivered vaginally) presented with a 4-month history of amenorrhea and a 2-day history of lower abdominal pain, vaginal bleeding, and increasing abdominal distension. She also reported easy fatigability, lightheadedness, and palpitations during the same period. Her previous antenatal care follow-ups and deliveries were in a health center where there was no ultrasound evaluation of pregnancy; hence, the bicornuate uterus had not been diagnosed previously. In her current pregnancy, the patient had not had any antenatal care visits until her current presentation.

She was alert but confused on physical examination, with a Glasgow Coma Scale score of 14/15 (E4M6V4). Her blood pressure was 100/60 mmHg and she exhibited tachycardia (pulse rate of 150 beats per minute). The conjunctiva and palms appeared pale. Abdominal examination revealed tenderness on palpation, with positive signs of fluid collection (fluid thrill and shifting dullness). A pelvic examination indicated a single closed cervix.

**Fig. 1:**
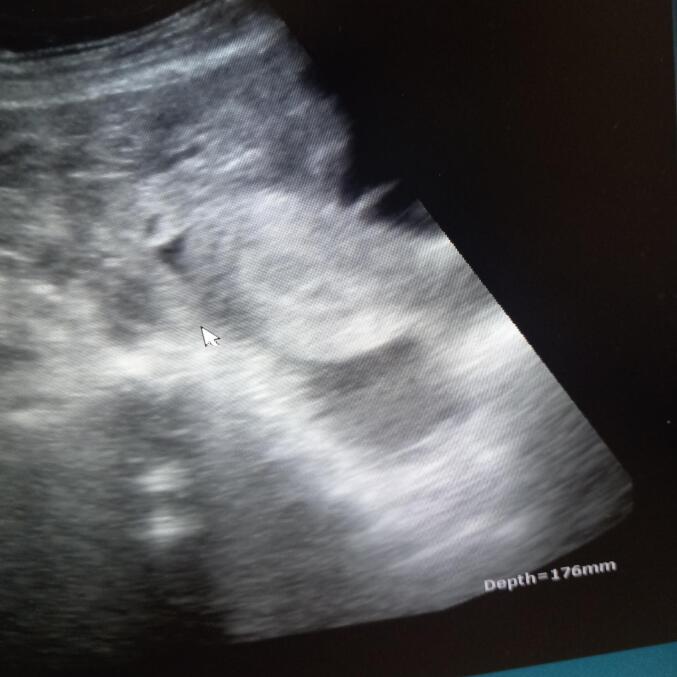
Empty uterus with fluid collection in the cul de sacs.

**Fig. 2:**
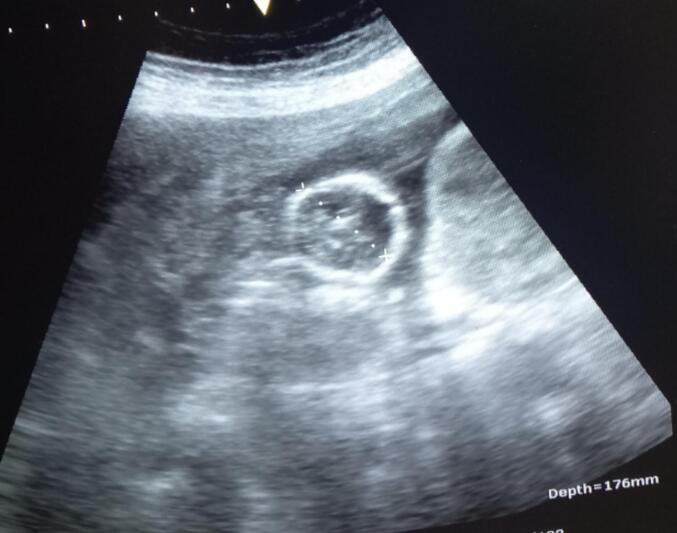
Fetal head in the peritoneal cavity; biparietal diameter of 16 weeks.

**Fig. 3:**
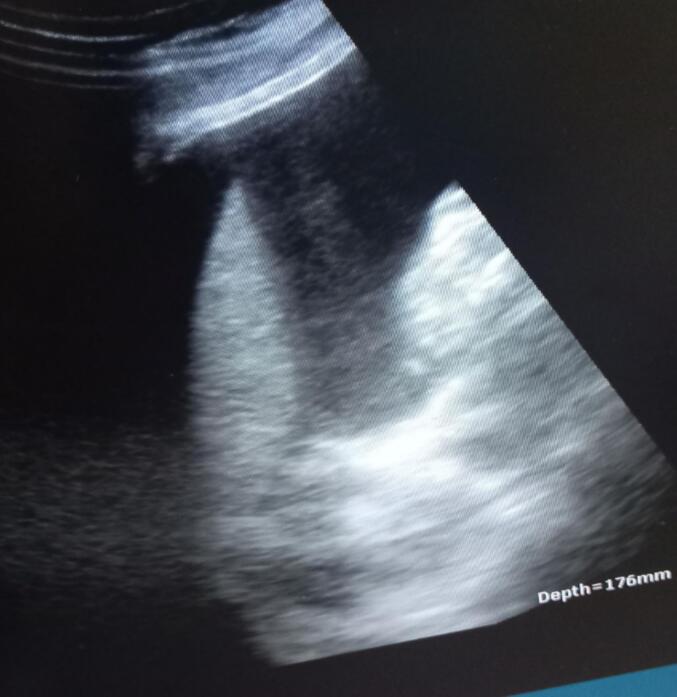
Excessive fluid collection in the Morrison's pouch.

Bedside ultrasound revealed an empty uterine cavity (Fig. 1) (the fluid tapped was bloody), a fetus of approximately 16 weeks by biparietal diameter, located outside the uterus (Fig. 2), as well as excessive fluid collection in the Morrison's pouch (Fig. 3).

Laboratory tests indicated hemoglobin of 2.9 g/dL, with normal platelet and white blood cell counts. Other investigations were unremarkable.

**Fig. 4:**
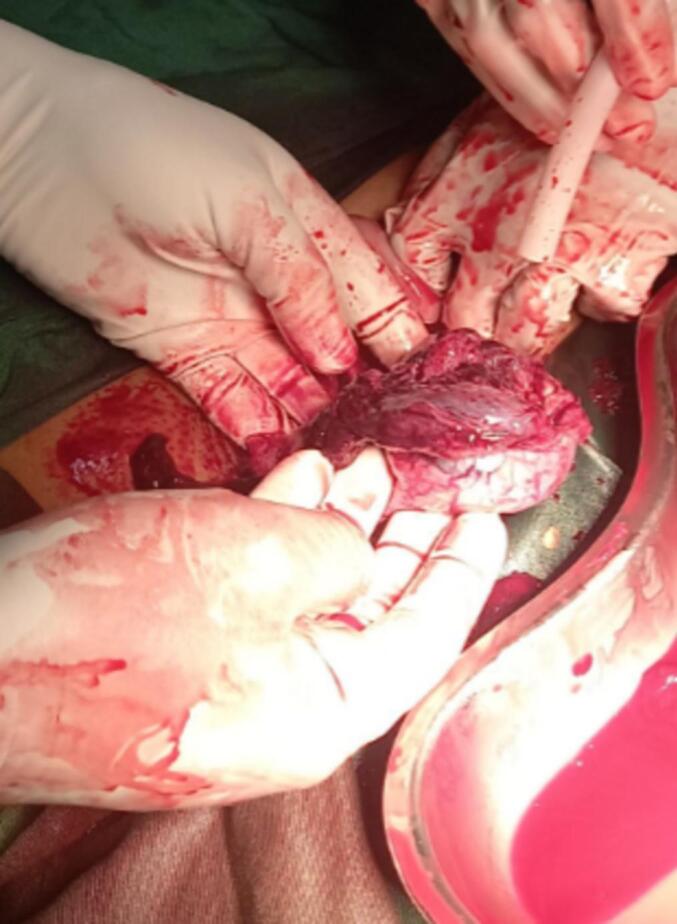
Ruptured fundus.

**Fig. 5:**
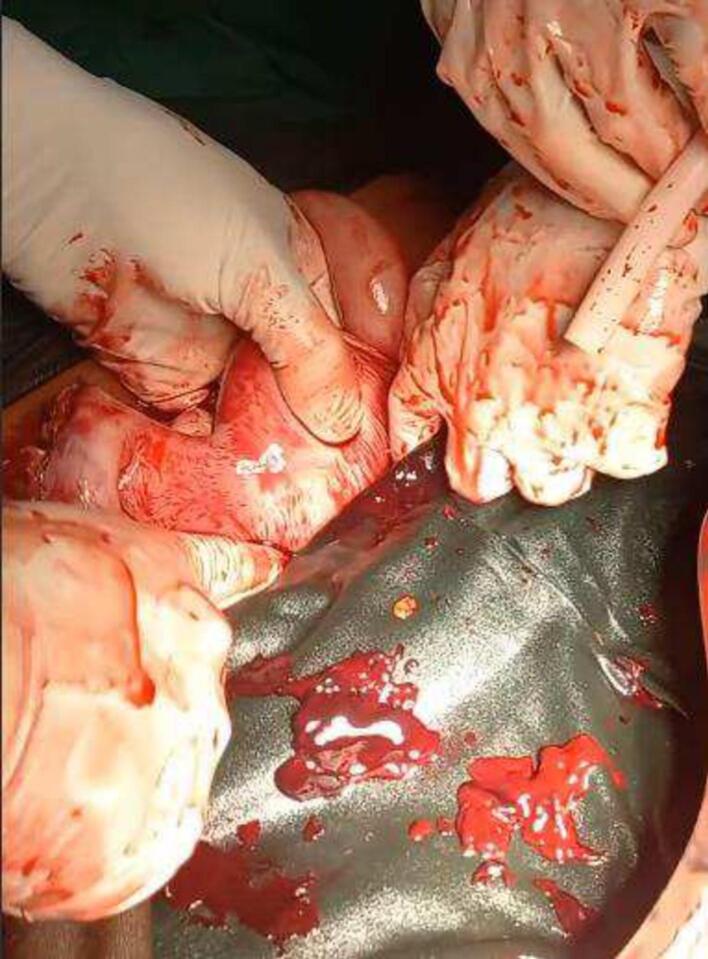
Bicornuate uterus, the ruptured and the normal uterine fundus.

She was taken to the operating room for exploratory laparotomy with a preoperative diagnosis of ruptured ectopic pregnancy. Intraoperatively, 4000 mL of blood was evacuated. There were two uterine horns with an indentation between them of >1 cm. The right uterine fundus had a communicating tract with the cervix and was found to be ruptured (Fig. 4), with the fetus floating in the peritoneal cavity and intact membranes. The left uterine fundus appeared normal (Fig. 5) along with bilateral adnexa, and the parametrium. A right hemihysterectomy was performed and the stump was sutured with Vicryl number 1 round. Otherwise, she had no other anomaly. The patient was admitted to the intensive care unit and received multiple transfusions of packed red blood cells. She was discharged in stable condition after counseling on family planning as she refused the permanent methods of contraceptive.

## 3 Discussion

Uterine rupture is a serious obstetric emergency that typically occurs during the third trimester or labor, but it can also manifest in the second trimester, particularly in the presence of uterine anomalies, with the usual site of the fundus [1,2]. Bicornuate uterus, a congenital malformation resulting from incomplete fusion of the Müllerian ducts, defined as a uterus with external indentation of >1.0 cm, presents unique challenges and risks during pregnancy. [4,5] This report describes a rare case of second-trimester uterine rupture in a patient with bicornuate uterus, leading to significant maternal morbidity due to massive hemoperitoneum.

Uterine rupture in the second trimester may be underrecognized [1,2]. In this case, the patient presented with classic signs of intra-abdominal bleeding, including lower abdominal pain, vaginal bleeding, and abdominal distension. The rapid deterioration of her clinical status emphasized the importance of maintaining a high index of suspicion for uterine rupture in patients with known uterine anomalies, especially when presenting with these symptoms.

Uterine anomalies such as bicornuate uterus are associated with an increased risk of obstetric complications, including miscarriage, preterm labor, and uterine rupture. The presence of two uterine corpora can lead to abnormal implantation or growth patterns, which may compromise the structural integrity of the uterus [3,4,6,7]. In this case, the rupture occurred at the right uterine fundus, which may have been weakened due to the anatomical variations associated with the bicornuate uterus.

The diagnosis of uterine rupture can be challenging due to its rarity and nonspecific presentation. [1] In this case, bedside ultrasound was pivotal in identifying the massive intraperitoneal fluid collection and confirming the absence of an intrauterine pregnancy. The prompt recognition of these findings facilitated timely surgical intervention. Exploratory laparotomy revealed a significant hemoperitoneum and a ruptured uterus with an intact fetus floating in the peritoneal cavity.

Management of uterine rupture in a bicornuate uterus typically involves surgical intervention to control hemorrhage and repair the uterine defect [1,6]. In the present case, a right hemihysterectomy was performed due to extensive damage to the right uterine horn.

## 4 Conclusion

This case highlights the critical importance of awareness regarding the potential for uterine rupture in patients with congenital anomalies like bicornuate uterus. Early recognition and prompt surgical intervention are vital in managing this life-threatening condition effectively. Continued education and vigilance among healthcare providers can help improve maternal outcomes in similar cases and ensure appropriate care for women with uterine anomalies during pregnancy. This could involve publishing and disseminating such rare case reports.
